# Genetic Deficiency of the Long Pentraxin 3 Affects Osteogenesis and Osteoclastogenesis in Homeostatic and Inflammatory Conditions

**DOI:** 10.3390/ijms242316648

**Published:** 2023-11-23

**Authors:** Valentina Granata, Dario Strina, Maria Lucia Schiavone, Barbara Bottazzi, Alberto Mantovani, Antonio Inforzato, Cristina Sobacchi

**Affiliations:** 1 IRCCS Humanitas Research Hospital, 20089 Rozzano, Italy; valentina.granata@humanitasresearch.it (V.G.); dario.strina@humanitasresearch.it (D.S.); marialucia.schiavone@humanitasresearch.it (M.L.S.); barbara.bottazzi@humanitasresearch.it (B.B.); alberto.mantovani@humanitasresearch.it (A.M.); 2 Milan Unit, Institute for Genetic and Biomedical Research (IRGB), National Research Council (CNR), 20138 Milan, Italy; 3 Department of Biomedical Sciences, Humanitas University, 20072 Pieve Emanuele, Italy; 4 The William Harvey Research Institute, Queen Mary University of London, London EC1M 6BQ, UK

**Keywords:** osteoblast, osteoclast, PTX3, bone, tissue remodeling, hyaluronan-rich matrix

## Abstract

The long pentraxin 3 (PTX3) is a soluble glycoprotein made by immune and nonimmune cells endowed with pleiotropic functions in innate immunity, inflammation, and tissue remodeling. PTX3 has recently emerged as a mediator of bone turnover in both physiological and pathological conditions, with direct and indirect effects on osteoblasts and osteoclasts. This notwithstanding, its role in bone biology, with major regard to the osteogenic potential of osteoblasts and their interplay with osteoclasts, is at present unclear. Here, we investigated the contribution of this pentraxin to bone deposition in the osteogenic lineage by assessing collagen production, mineralization capacity, osteoblast maturation, extracellular matrix gene expression, and inflammatory mediators’ production in primary osteoblasts from the calvaria of wild-type (WT) and *Ptx3*-deficient (*Ptx3^−/−^*) mice. Also, we evaluated the effect of PTX3 on osteoclastogenesis in cocultures of primary osteoblasts and bone marrow-derived osteoclasts. Our investigations were carried out both in physiological and inflammatory conditions to recapitulate in vitro aspects of inflammatory diseases of the bone. We found that primary osteoblasts from WT animals constitutively expressed low levels of the protein in osteogenic noninflammatory conditions, and genetic ablation of PTX3 in these cells had no major impact on collagen and hydroxyapatite deposition. However, *Ptx3^−/−^* osteoblasts had an increased RANKL/OPG ratio and CD44 expression, which resulted in in enhanced osteoclastogenesis when cocultured with bone marrow monocytes. Inflammation (modelled through administration of tumor necrosis factor-α, TNF-α) boosted the expression and accumulation of PTX3 and inflammatory mediators in WT osteoblasts. In these conditions, *Ptx3* genetic depletion was associated with reduced collagen deposition and immune modulators’ production. Our study shed light on the role of PTX3 in osteoblast and osteoclast biology and identified a major effect of inflammation on the bone-related properties of this pentraxin, which might be relevant for therapeutic and/or diagnostic purposes in musculoskeletal pathology.

## 1. Introduction

Pentraxin 3 (PTX3) is a soluble glycoprotein of the pentraxin superfamily, first identified in the early 1990s in endothelial cells, that is made by a variety of cell types, including fibroblasts, adipocytes, mesenchymal stromal cells, and smooth muscle cells, in response to several stimuli (e.g., interleukin-1beta, IL-1β; tumor necrosis factor-alpha, TNF-α; other proinflammatory signals and Toll-like receptor engagement) [1]. PTX3 exerts pleiotropic functions [1,2,3], possibly as a reflection of a rather unique molecular structure [4,5]. The current literature indicates that it acts as a soluble pattern recognition molecule (PRM); upon binding to pathogen-associated molecular patterns (PAMPs), it coordinates efficient immune responses through the activation and regulation of the complement cascade and facilitation of pathogen recognition by phagocytes [1].

An increasing body of evidence points to this pentraxin as a key player in extracellular matrix biology in addition to innate immunity. In this regard, PTX3 is known to interact with the hyaluronic acid (HA)-binding protein tumor necrosis factor-stimulated gene 6 (TSG-6) and the heavy chains (HCs) of the serum proteoglycan inter-alpha-trypsin inhibitor (IαI) to assemble and stabilize functionally competent HA-rich matrices. This is essential for the formation and expansion of the cumulus oophorus complex, and thereby for female fertility [6]. In addition, PTX3 exerts pivotal functions in tissue remodeling and repair [7]. In this regard, the acidification of the extracellular milieu that accompanies wound healing was reported to promote the binding of PTX3 to fibrinogen, fibrin, and plasminogen and favor pericellular fibrinolysis [7].

Moreover, PTX3 has emerged as an important player in bone biology [3]. Gene inactivation in the mouse led to reduced trabecular bone volume during physiological development and delayed callus mineralization in a tibial fracture model, possibly due to PTX3 being able to sequester fibroblast growth factor 2 (FGF2) and restrain the inhibitory effects of this factor on bone formation [8]. In other settings, pathogenetic roles have emerged for this protein. For example, in a mouse model of osteoarthritis, PTX3 deficiency significantly reduced the severity of the disease by alleviating cartilage degeneration and systemic inflammation [9]. Also, in humans, PTX3 levels have been associated with various bone-loss conditions such as arthritis, periodontal tissue inflammation, osteolytic bone metastases, bone infection, and bone repair [10,11,12,13]. Amongst skeletal cells, those of the osteogenic lineage express PTX3, whereas production of this protein has never been documented in osteoclasts. Lee and colleagues showed that human preosteoblasts (pre-OBs, defined as bone marrow-derived stromal cells (BMSCs) cultured in osteogenic induction medium (oim) for 1 week) make high levels of PTX3, which were further amplified when these cells were treated with TNF-α for 24 h. As opposed to pre-OBs, mature OBs (i.e., BMSCs in oim for 3 weeks) were poor producers of the protein [14]. Administration of exogenous PTX3 to mouse pre-OBs did not affect osteoblastogenesis; however, it enhanced runt-related transcription factor 2 (*Runx2*) expression and increased the Receptor Activator Of Nuclear Factor Kappa B Ligand/Osteoprotegerin (RANKL/OPG) ratio. In line with this, the addition of the recombinant protein to cocultures of pre-OBs and bone marrow-derived osteoclast precursors promoted the formation of Tartrate-Resistant Acid Phosphatase (TRAP)^+^ cells. In this experimental setting, small interfering RNA (siRNA)-induced downregulation of *Ptx3* expression caused a reduction in the RANKL/OPG ratio and partially abolished TNF-α-dependent osteoclastogenesis [14]. Accordingly, PTX3 expression was reported to be elevated in bone metastases of breast cancer, whereby this pentraxin has been proposed to be involved in the inflammation-associated osteolytic complications of breast cancer [15].

In contrast with the findings by Lee et al. [14], Scimeca and colleagues observed a positive effect of PTX3 on osteoblastogenesis. In fact, these authors documented a reduced expression of PTX3 (in terms of the PTX3^+^ area in immunohistochemical images of femoral head biopsies) in osteoporotic individuals compared to patients affected by osteoarthritis and healthy young subjects [16]. In their study, human primary OBs from healthy individuals treated with an anti-PTX3 antibody displayed an altered morphology with reduced OB gene expression and formed fewer hydroxyapatite microcrystals. Conversely, primary OBs from osteoporotic patients treated with exogenous PTX3 had higher rates of proliferation and hydroxyapatite microcrystal formation. More recently, Liu and colleagues documented PTX3 expression in MC3T3-E1 cells in basal conditions as well as during osteogenic induction [17]. They also showed that overexpression of *Ptx3* by lentiviral transduction during osteogenic induction increased OB gene expression and OB formation and highlighted the involvement of the PI3K/Akt axis as a downstream signaling pathway. Moreover, Dong and colleagues reported that MC3T3-E1 cultured in osteogenic conditions in the presence of TNF-α had increased production of PTX3 and reduced expression of *Runx2*, *Sp7*, and bone gamma-carboxyglutamate protein (*Bglap*) and alkaline phosphatase (ALP) activity. Conversely, Ptx3 overexpression overcame the inhibitory effects of the TNF-α treatment on the osteogenic potential of these cells [18]. Of note, PTX3 was found in the HA-rich pericellular matrix and was demonstrated to promote HA synthesis and CD44 expression and their interaction. This led to the activation of the FAK/Akt signaling cascade and expression of the *HA synthases 1/2/3* and *Cd44* in a positive feedback loop.

These conflicting reports on the role of PTX3 in bone biology are likely due to intrinsic limitations of the applied experimental settings that hinged upon the virally mediated overexpression or downregulation of the gene (with siRNA or short hairpin (sh)RNA technology), exogenous administration of the protein (or blocking antibodies), and the use of osteoblastic lineage cells (or cell lines) with varying degrees of differentiation and maturation.

Here, we investigated the functions of PTX3 in OB biology by undertaking a genetic approach based on osteogenic lineage cells isolated from PTX3-deficient mice (*Ptx3^−/−^*). In this regard, we established primary OB mono- and OB-osteoclast (OC) cocultures and evaluated collagen production, mineralization capacity, osteogenic gene expression, RANKL/OPG ratio, and osteoclastogenic potential. Moreover, to recapitulate in vitro aspects of inflammatory diseases of the bone, we assessed the response of *Ptx3^−/−^* OBs to TNF-α stimulation as a prototype of inflammatory cytokines.

## 2. Results

### 2.1. Effect of Ptx3 Genetic Deficiency on Osteogenic Functions of Primary OBs

To address the roles of PTX3 in OB biology, we established in vitro cultures of primary OBs isolated from the calvariae of WT and *Ptx3^−/−^* mice according to a standard procedure (see Section 4). These cells can be regarded as pre-OBs in that they are committed to becoming mature OBs but still lack full osteogenic competence; we analyzed them in the basal condition and at different timepoints during osteogenesis [19].

**Figure 1:**
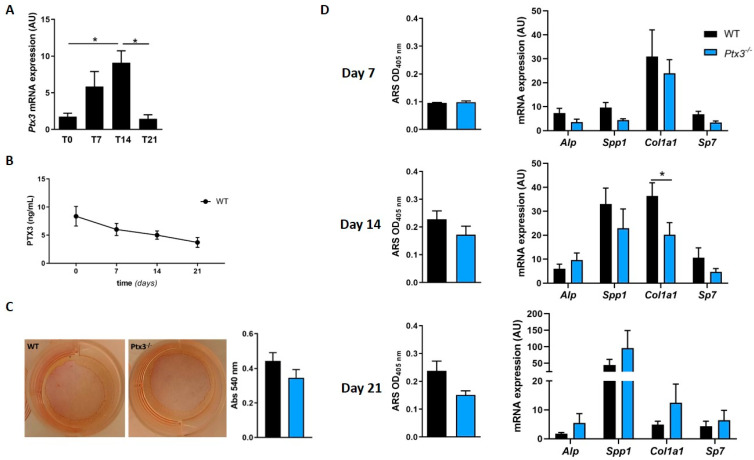
Effect of *Ptx3* genetic deficiency on the osteogenic properties of OBs. (**A**) Analysis of Ptx3 expression in primary OBs from calvariae of WT mice in basal condition and at different timepoints during osteogenic induction. *Ptx3* mRNA levels were determined by qPCR. Results were normalized based on *Gapdh* expression and reported as arbitrary units (AU); * *p* < 0.05. (**B**) PTX3 protein was quantified in the supernatant of WT OB cultures by ELISA (3 independent experiments performed in duplicate, *n* = 6, mean ± SEM). One-way ANOVA with multiple comparison test. (**C**) Assessment of in vitro collagen production by WT and *Ptx3^−/−^* OBs stimulated with ascorbic acid for 8 h. Cultures were stained with Sirius red; representative images are shown. Afterwards, staining was extracted, and absorbance measured at 540 nm. Mann–Whitney test. (**D**) Time-course analysis of mineralization in WT and *Ptx3^−/−^* primary OB cultures, as assessed by Alizarin red staining (ARS) and quantification at 405 nm and expression of osteogenic marker genes. One-way ANOVA with multiple comparison test; * *p* < 0.05. All the data presented in this figure are derived from 3 independent experiments performed in duplicate, *n* = 6, and are represented as mean ± SEM.

First, we measured *Ptx3* expression in WT OBs in homeostatic condition (t = 0) and upon osteogenic induction. We found that *Ptx3* was transcribed in WT pre-OBs, and the corresponding mRNA steadily increased with time, with a peak at 14 days of osteogenic induction (Figure 1A), and then dropped to baseline levels at 21 days. 

The concentration of the PTX3 protein in the supernatant of primary OB cultures was 8.36 ± 1.74 ng/mL at t = 0 and remained stable during mineralization (7 to 14 days), with a trend (not significant) of reduction at 21 days (Figure 1B).

To assess the effect(s) of PTX3 on the osteogenic potential of OBs, we evaluated collagen deposition by WT and *Ptx3^−/−^* OBs upon stimulation with ascorbic acid. Sirius red staining (which is selective for collagen fibers) showed no difference between OBs isolated from the two genotypes (Figure 1C, absorbance at 540 nm: 0.44 ± 0.047 for the WT vs. 0.35 ± 0.048 for *Ptx3^−/−^*). We then analyzed the mineralization capacity at 7, 14, and 21 days of osteogenic induction and found that WT and *Ptx3^−/−^* OBs deposited comparable amounts of hydroxyapatite as assessed by Alizarin red staining (ARS; Figure 1D, left column). Accordingly, no differences were found in the expression of genes that are involved in OB maturation (e.g., *Alp*; secreted phosphoprotein 1, *Spp1*, encoding the matrix protein osteopontin; Sp7 transcription factor, *Sp7*, encoding the transcription factor osterix) with the only exception being collagen type I alpha 1 chain, *Col1a1*, which was significantly lower in mature *Ptx3^−/−^* OBs compared to their WT counterparts at 14 days (Figure 1D, right column).

Overall, genetic ablation of PTX3 was associated with a trend (though not significant) of reduced osteogenic potential in primary OBs and a clearcut effect on *Col1a1* expression.

### 2.2. Effect of Ptx3 Genetic Deficiency on the Osteoclastogenic Potential of Primary OBs

In the bone, OBs have an intense crosstalk with OC progenitors and mature OCs. This allows OBs to regulate OC differentiation, survival, and function, either through direct cell-to-cell contacts or by means of secreted factors, among which RANKL, OPG, and Macrophage Colony-Stimulating Factor (M-CSF) are key players [20]. M-CSF promotes OC progenitor survival and expression of RANK, a functional receptor of the essential osteoclastogenic factor RANKL. Conversely, OPG is the decoy receptor for RANKL and impedes its binding to RANK and the activation of the downstream signalling pathway; this leads to the inhibition of OC formation and activation. In fact, the RANKL/OPG ratio is commonly used as an indicator of the OB/OC activity balance.

**Figure 2:**
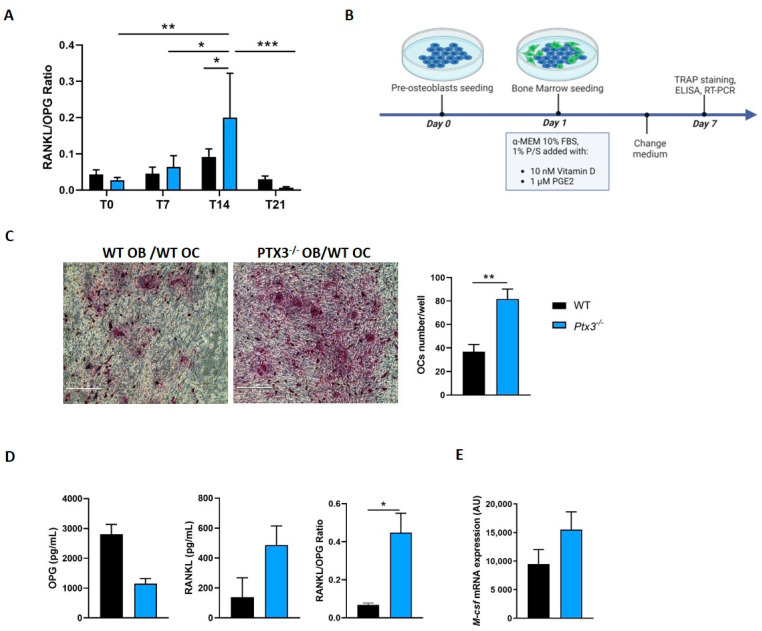
Effect of *Ptx3* genetic deficiency on OB-induced osteoclastogenesis. (**A**) RANKL/OPG ratio in the supernatant of WT and *Ptx3^−/−^* OB cultures in basal conditions and at different timepoints of osteogenic induction. RANKL and OPG were quantified using dedicated ELISA kits (3 independent experiments performed in duplicate, *n* = 6, mean ± SEM). Two-way ANOVA with multiple comparison test; * *p* < 0.05, ** *p* < 0.01, *** *p* < 0.001. (**B**) Schematic representation of the protocol for coculture experiments. (**C**) Representative images of TRAP-stained OCs formed after coculture of WT or *Ptx3^−/−^* OBs with WT bone marrow cells for 7 days. Images were taken with an EVOS microscope at 20× magnification; scale bar: 200 μm. TRAP^+^ multinucleated (nuclei number ≥ 3) OCs were counted by a blind operator and expressed as number of mature OCs per well. Mann–Whitney test; ** *p* < 0.01. (**D**) RANKL, OPG, and RANKL/OPG ratio in the supernatant of the cocultures described in C. The conditioned medium was collected from each well after 7 days of coculture (2 independent experiments performed in triplicate, *n* = 6, mean ± SEM). Mann–Whitney test; * *p* < 0.05. (**E**) Analysis of *M-csf* expression in the coculture setting described in (**C**) after 7 days of coculture; mRNA levels were determined by qPCR. Results were normalized based on *Gapdh* expression and expressed as arbitrary units (AU). Mann–Whitney test.

If and how PTX3 affects the OB/OC crosstalk has been the subject of previous investigations with contrasting outcomes [14,15,16,17,18]. To clarify this aspect, here, we evaluated the impact of *Ptx3* genetic deficiency on the OB osteoclastogenic potential. First, we quantified RANKL and OPG in the supernatant of WT and *Ptx3^−/−^* OBs at baseline and during osteogenic induction using high-sensitivity ELISA assays. The RANKL/OPG ratio did not differ between the two genotypes in cultures of pre-OBs and early OBs, while it was significantly higher in the supernatant of mature *Ptx3^−/−^* OBs compared to WT at 14 days of osteogenic induction (Figure 2A).

Prompted by these observations, we evaluated whether *Ptx3^−/−^* OBs had a higher osteoclastogenic potential compared to WT. To this end, we established cocultures of either *Ptx3^−/−^* or WT mature OBs and WT OC progenitors. It is worth noting here that, as opposed to OBs, mature OCs do not make PTX3, and synthesis of the protein is confined to OC progenitors only (e.g., monocytes) in inflammatory conditions [3]. In our setting, bone marrow cells from WT mice were seeded on top of either *Ptx3^−/−^* or WT mature OBs in OB medium containing vitamin D and PGE2, which are known to stimulate production of the essential osteoclastogenic factors by OBs (Figure 2B). After 7 days of coculture, OC formation was evaluated by means of TRAP staining followed by the counting of TRAP^+^ cells (bona fide, OCs). We found that a larger number of OCs formed in the presence of *Ptx3^−/−^* OBs (Figure 2C). Accordingly, RANKL was higher and OPG lower in the supernatant of cocultures with *Ptx3^−/−^* OBs, which resulted in higher RANKL/OPG ratios in this setting (Figure 2D). Along the same lines, the *M-csf* gene had a trend of increased transcription in *Ptx3^−/−^* when compared to WT OBs (Figure 2E).

Overall, these data indicated that *Ptx3^−/−^* OBs had enhanced osteoclastogenic potential.

### 2.3. Effect of Ptx3 Genetic Deficiency on the Expression of OB Membrane Markers and Genes Involved in HA Matrix Synthesis and Assembly

The interaction of OBs (in their various differentiation stages) with the bone extracellular matrix is crucial for OB differentiation, survival, proliferation, and matrix mineralization. Given that PTX3 is an important component of the extracellular matrix and participates in its remodeling [7], we asked whether *Ptx3* genetic deficiency could impact this aspect of the OB biology.

**Figure 3:**
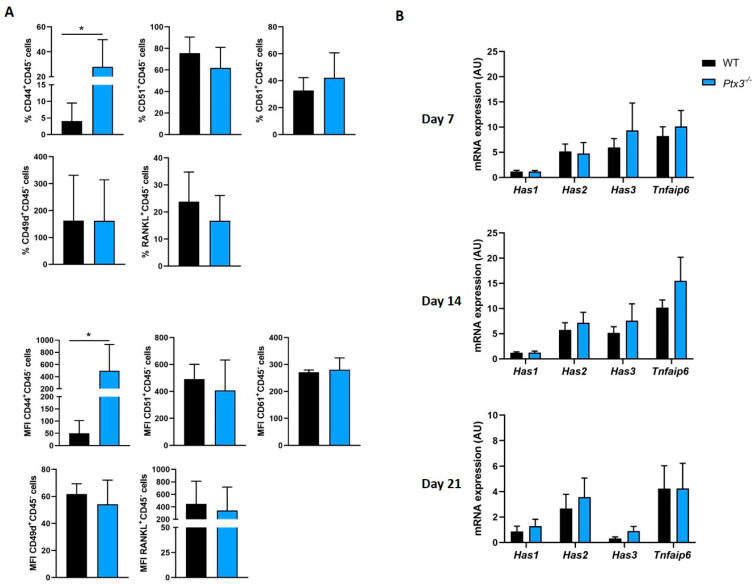
(**A**) FACS analysis of the indicated surface markers on WT and *Ptx3^−/−^* KO OBs in basal condition (not treated with oim). The percentage of CD45^-^ cells expressing each marker as well as the mean fluorescence intensity for each marker in both genotypes is indicated as mean ± SEM. Mann–Whitney test; * *p* < 0.05. (**B**) Gene expression analysis of the hyaluronic acid synthases *Has1*, *Has2*, and *Has3* and *Tnfaip6* gene in WT and *Ptx3^−/−^* OBs at different timepoints during osteogenic induction; mRNA levels were determined by qPCR and normalized on *Gapdh* expression, and they are indicated as arbitrary units (AU) (3 independent experiments performed in duplicate, *n* = 6, mean ± SEM). One-way ANOVA.

To this end, we assessed the expression of selected surface markers that are known to be involved in cell–matrix and cell–cell interactions, namely, CD44, which acts as a receptor for HA, osteopontin, collagens, and PTX3 [18,21]; CD51, also known as integrin subunit alpha V, and CD61, or integrin beta chain 3, which recognize the primary amino acid sequence RGD in a wide array of ligands like vitronectin, fibronectin, fibrinogen, laminin, osteopontin, prothrombin, thrombospondin, and von Willebrand factor [22]; CD49d, alias integrin subunit alpha 4, which associates with beta subunit integrins and recognizes fibronectin, VCAM1, and other ligands [22]; and RANKL, which is primarily expressed in the membrane-bound form [23]. These evaluations were carried out by means of flow cytometry on WT and *Ptx3^−/−^* OBs cultured in osteogenic medium. We found that while the cellular levels of CD51, CD61, CD49d, and RANKL did not differ across the two genotypes, more CD44^+^ cells and a higher expression of this marker were observed in *Ptx3^−/−^* OBs compared to their WT counterparts (Figure 3A). 

Based on previous work [18], we speculate that this is due to a compensatory mechanism. In fact, in the absence of PTX3, the HA extracellular network loses stability and affinity/avidity for its cognate cellular receptor CD44, a process that could be counteracted, at least in part, by enhanced surface expression of CD44. This hypothesis is corroborated by the observation that genetic deficiency of PTX3 did not affect the expression of genes involved in HA synthesis (*Has1*,*2*,*3*) and crosslinking (*Tnfaip6*, which encodes TSG-6) (Figure 3B).

### 2.4. Effect of Ptx3 Genetic Deficiency on the Expression of Immune Mediators by OB

**Figure 4:**
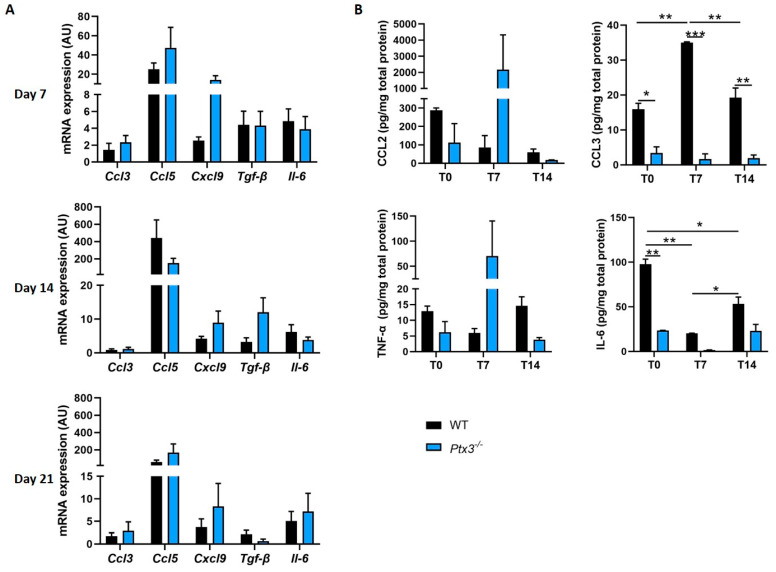
Expression of immune mediators in WT and *Ptx3^−/−^* KO OBs at different timepoints during osteogenic induction. (**A**) Gene expression analysis of selected factors (as indicated in the graphs) known to attract and regulate both immune cells and skeletal cells and their progenitors; mRNA levels were determined by qPCR and normalized on *Gapdh* expression, and they are indicated as arbitrary units (AU) (3 independent experiments performed in duplicate, *n* = 6, mean ± SEM). One-way ANOVA. (**B**) Protein expression of selected factors (as indicated in the graphs) in cell lysates of WT and *Ptx3^−/−^* OBs. Protein levels were measured using a highly sensitive immunoassay and normalized on the total amount of proteins in the cell lysates (*n* ≥ 3, mean ± SEM). Two-way ANOVA. * *p* < 0.05, ** *p* < 0.01, *** *p* < 0.001.

OBs are known to produce soluble factors such as cytokines and chemokines, which act as chemoattractant cues and regulators not only of immune cells but also of skeletal cells and their progenitors [20]. Building upon the knowledge that PTX3 is a key humoral effector of innate immunity that actively participates in immune homeostasis [1], we wondered whether this pentraxin could also be involved in the functions of OBs as nonprofessional immune cells. To this end, we evaluated the expression of selected inflammatory mediators, including C-C motif chemokine ligand 3 and 5 (*Ccl3* and *Ccl5*), which have a dual role in bone metabolism, enhancing osteoclastogenesis, and reducing osteoblastogenesis; C-X-C motif chemokine ligand 9 (*Cxcl9*), which is constitutively expressed by OBs and controls their proliferation, differentiation, and mineralization [24]; *Tgf-β*, whose protein product modulates bone formation and resorption and is largely buried in the matrix [25]; and *Il-6*, which enhances OC formation and modulates immune cells [20]. We found a similar pattern of expression for *Ccl3*, *Ccl5*, *Tgf-β*, and *Il-6* in WT and *Ptx3^−/−^* OBs during osteogenic induction and a trend to higher levels of *Cxcl9* mRNA in *Ptx3^−/−^* OBs at 7 days postinduction only, which, however, were comparable to those observed in WT cells when OBs became fully mature (days 14 and 21 in Figure 4A).

We also measured the protein concentration of key inflammatory mediators (namely, CCL2, CCL3, IL-6, and TNF-α) in the cell homogenate of primary OBs at different stages of maturation (0, 7, and 14 days of osteogenic induction). We found that the levels of CCL3 were lower in *Ptx3^−/−^* than WT OBs at all timepoints, despite the fact that the amount of the corresponding transcript was not affected by genetic depletion of PTX3. IL-6 was reduced in *Ptx3^−/−^* OBs at the pre-OB stage only (t = 0). No differences were observed across the two genotypes in the levels of CCL2 and TNF-α, although *Ptx3^−/−^* OBs had a trend of increasing concentration of both mediators at 7 days from osteogenic induction, which was not seen in WT OBs (Figure 4B).

### 2.5. Effect of Ptx3 Genetic Deficiency on OB Functions in Inflammation

Inflammatory mediators are required for the structural integrity and metabolic balance of the bone during physiological remodeling; nonetheless, excessive and uncontrolled inflammation is known to have detrimental effects on this tissue. In humans, the levels of the PTX3 protein have been associated with various inflammatory diseases of the bone [10,11,12,13]. Several studies, mostly in vitro, were conducted to elucidate the role of this pentraxin in the response of bone cells to inflammatory stimuli and led to contrasting outcomes [14,15,16,17,18]. To address this issue, here, we assessed the effect of PTX3 genetic depletion on major OB functions in the presence of TNF-α, a prototypic inflammatory cytokine that is known to enhance PTX3 expression in these cells.

**Figure 5:**
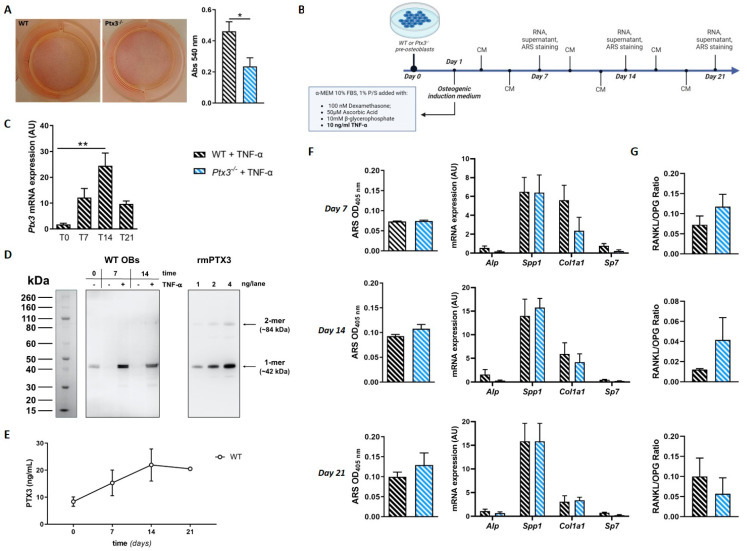
Effect of *Ptx3* genetic deficiency on OB functions in the presence of inflammation. (**A**) Assessment of in vitro collagen production by WT and *Ptx3^−/−^* OBs treated with TNF-α for 24 h prior to stimulation with ascorbic acid. Cultures were stained with Sirius red; representative images are shown here. Afterwards, staining was extracted, and absorbance measured at 540 nm. Mann–Whitney test; * *p* < 0.05. (**B**) Schematic representation of the experimental protocol for osteogenic induction in the presence of TNF-α. CM = conditioned medium. (**C**) Analysis of *Ptx3* expression in WT OBs in basal condition and at different timepoints during osteogenic induction in the presence of TNF-α. One-way ANOVA with multiple comparison test; ** *p* < 0.01. (**D**) WT OBs were lysed at 0, 7, and 14 days of osteogenic induction in the absence and presence of TNF-α. Lysate samples containing 10 μg total proteins were analyzed by Western blotting using an anti-mouse PTX3 polyclonal antibody. Purified recombinant mouse PTX3 (rmPTX3) was used as a reference (1, 2, and 4 ng/lane). A representative gel is shown with indication of the apparent molecular weight of the protein standards assigned following the manufacturer’s instructions for NuPAGE Bis-Tris 4–12% with MES buffer (Life Technologies). In these gels, PTX3 is detected as two immune reactive bands with apparent molecular weights of 42 (1-mer) and 84 (2-mer) kDa [4]. (**E**) PTX3 protein in the supernatant of WT OB cultures treated with TNF-α during osteogenic induction was quantified by ELISA. One-way ANOVA with multiple comparison test. (**F**) Time-course analysis of mineralization and expression of osteogenic marker genes by WT and *Ptx3^−/−^* KO primary OB cultures in the presence of TNF-α. (**G**) Time-course analysis of RANKL/OPG ratio in the supernatant of WT and *Ptx3^−/−^* KO primary OB cultures in the presence of TNF-α during osteogenic induction. All the data presented in this figure (except Western blot) are derived from 3 independent experiments performed in duplicate, *n* = 6, and are represented as mean ± SEM.

First, we measured collagen production by WT and *Ptx3^−/−^* OBs pretreated for 24 h with 10 ng/mL of TNF-α, in line with published protocols [14]. Sirius red staining showed significantly lower collagen production by *Ptx3^−/−^* OBs compared to their WT counterparts (Figure 5A).

Then, we evaluated the effect of TNF-α stimulation during osteogenic induction (the applied experimental protocol is depicted in Figure 5B and detailed in the Section 4). In these conditions, in WT OBs, *Ptx3* expression had a similar trend to that observed in the physiological setting with a significant increase in the corresponding mRNA levels at 14 days of osteogenic induction compared to day 0. In addition, at all timepoints, *Ptx3* mRNA was more abundant in the presence than in the absence of TNF-α with a statistically significant difference at 14 days of osteogenic induction (WT: 9.09 ± 1.63 AU vs. WT + TNF-α: 24.4 ± 4.96, *p* < 0.0001; Figure 1A and Figure 5C for a comparison).

At the protein level, PTX3 expression (assessed by Western blot analysis of cell homogenates) was markedly increased by TNF-α compared to baseline levels at 7 and 14 days of osteogenic induction (Figure 5D). Also, at these timepoints and in the absence of the inflammatory cytokine, the protein was barely detectable and clearly less abundant than at day 0, consistent with the trend of decreasing PTX3 concentration observed in the condition medium of WT OBs during osteogenesis (see Figure 1B).

PTX3 concentration in the culture medium of WT OBs increased with time, with a maximum at 14 days of osteogenic induction (21.93 ± 5.94 ng/mL; Figure 5E). Furthermore, at all timepoints it was higher than that observed in the absence of TNF-α (*p* < 0.01; see Figure 1A for comparison), consistent with previous reports [14].

ARS staining at weekly intervals during osteogenic induction in the presence of TNF-α did not show differences in the mineralization capacity between WT and *Ptx3^−/−^* OBs (Figure 5F), however, at 14 and 21 days of osteogenic induction, the mineral content of the newly deposited matrix in WT OBs was lower in the presence than in the absence of TNF-α (absorbance at 405 nm: WT: 0.23 ± 0.03 vs. WT + TNF-α: 0.09 ± 0.003, *p* = 0.017, at day 14; WT: 0.24 ± 0.034 vs. WT + TNF-α: 0.1 ± 0.012, *p* = 0.013, at day 21; see Figure 1C for a comparison). Expression of selected osteogenic genes (*Alp*, *Spp1*, *Sp7*, *Col1a1*) was no different between WT and *Ptx3^−/−^* OBs (Figure 5F), and lower levels of the corresponding mRNA were observed in the presence (compared to the absence) of TNF-α regardless of the OB genotype (see Figure 1D for a comparison). Finally, in these experimental conditions, no significant difference was observed in the RANKL/OPG ratio between WT and *Ptx3^−/−^* OBs (Figure 5G).

**Figure 6:**
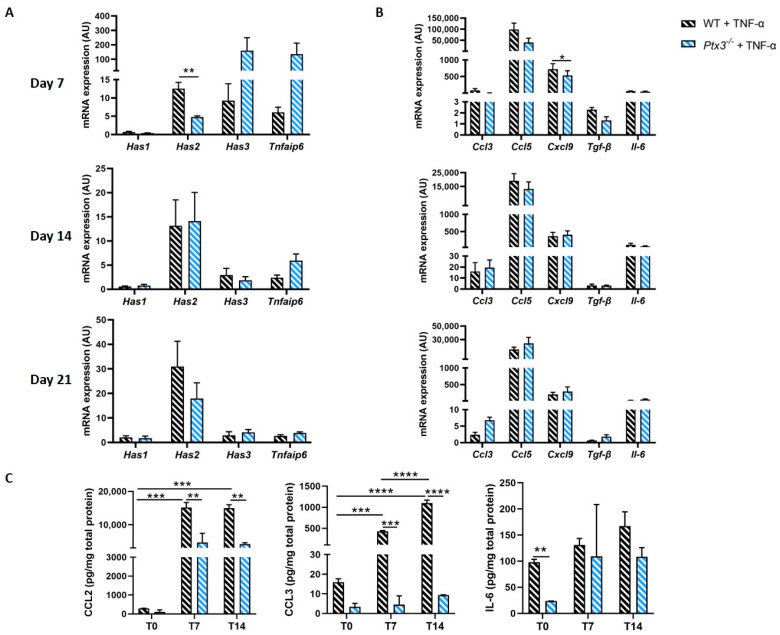
(**A**) Expression of *Ha synthase* isoforms and *Tnfaip6* in WT and *Ptx3^−/−^* KO OBs at different timepoints during osteogenic induction in inflammatory condition. (**B**) Gene expression analysis of selected immune regulatory factors (as indicated in the graphs). In (**A**,**B**), mRNA levels were determined by qPCR and normalized on *Gapdh* expression, and they are indicated as arbitrary units (AU) (3 independent experiments performed in duplicate, *n* = 6, mean ± SEM). One-way ANOVA. (**C**) Protein expression of selected factors (as indicated in the graphs) in cell lysates of WT and *Ptx3^−/−^* OBs. Protein levels were measured by immunoassay and normalized on the total amount of proteins in the cell lysates (*n* ≥ 3, mean ± SEM). Statistical analysis using two-way ANOVA. * *p* < 0.05, ** *p* < 0.01, *** *p* < 0.001, **** *p* < 0.0001.

PTX3 was proposed to promote expression of the HA-making genes *Has1/2/3* through a positive feedback loop [18]. In our hands, *Has1* expression upon stimulation with TNF-α did not change compared to that in basal conditions and did not differ between WT and *Ptx3^−/−^* OBs. Also, TNF-α induced *Has2* overexpression, particularly after 21 days of osteogenic induction, and *Has2* mRNA levels were higher in WT than in *Ptx3^−/−^* OBs at 7 days. Of note, TNF-α did not boost expression of *Has3* and *Tnfaip6*; however, a trend of increased transcription was observed for both genes in the *Ptx3^−/−^* OBs at 7 days of osteogenic induction (Figure 6A). 

The expression levels of all tested cytokines and chemokines increased both in WT and *Ptx3^−/−^* OBs upon TNF-α treatment, and only *Cxcl9* differed between the two genotypes, with significantly lower expression in *Ptx3^−/−^* vs. WT OBs at 7 days of osteogenic induction (Figure 6B).

At the protein level, TNF-α stimulation significantly increased CCL2 and CCL3 in WT OBs at 7 and 14 days of osteogenic induction. Furthermore, at these timepoints, the concentration of both chemokines was significantly lower in *Ptx3^−/−^* than in WT OBs. Finally, the levels of IL-6 did not clearly change during inflammatory osteogenesis, although *Ptx3^−/−^* pre-OBs made more of this cytokine prior to osteogenic induction, i.e., at day 0 (Figure 6C).

## 3. Discussion

Bone remodeling is a highly coordinated process that hinges upon the cooperative action of specialized cells of mesenchymal and hematopoietic origin (i.e., osteoblasts and osteoclasts, respectively) and additional contributions from immune cells [26,27,28,29,30]. This process is mediated by the essential players RANKL, OPG, M-CSF, and their cognate receptors and finely regulated by several additional factors including hormones, extracellular matrix components, and inflammatory molecules [31,32,33,34,35,36]. The long pentraxin PTX3 is a soluble pattern recognition molecule (PRM) with pleiotropic functions in inflammation, innate immunity, and tissue repair and emerging roles in bone pathophysiology [1,37,38]. PTX3 expression was documented in osteogenic lineage cells both in vivo and in vitro, and no evidence is available to support production of this PRM by mature OCs [3]. The locally made protein has been implicated in several physiological and pathological processes of the bone microenvironment; however, how it affects the osteogenic potential of osteoblasts and their interplay with osteoclasts is still a matter of debate.

Here, we addressed this point by implementing a genetic approach based on primary OBs isolated from *Ptx3^−/−^* and WT mice. First, we documented that PTX3 is expressed by murine pre-OBs both in basal and osteogenic conditions, and TNF-α stimulation (that recapitulates aspects of chronic inflammation of the bone) augmented synthesis and release of the protein, in agreement with the notion that this PRM acts as a local inflammatory gene [3].

We then assessed the roles of this pentraxin in the osteogenic functions of OBs. Of note, recombinant formulations of PTX3 have been shown to promote OB differentiation and calcification in primary human OB cultures from healthy donors and osteoporotic patients [38]. Also, pharmacological blockade of the endogenous protein (with a monoclonal antibody) was reported to impair proliferation and hydroxyapatite microcrystal formation in human primary OBs [17]. Conversely, the addition of exogenous PTX3 to murine calvaria OB precursor cells in osteogenic medium (an experimental setting resembling that used in this study) did not affect OB differentiation nor did it have osteogenic effects [14]. In our experimental setting, we observed a trend of reduced collagen production and matrix mineralization in the absence of PTX3 (i.e., in *Ptx3^−/−^* OBs) in homeostatic (i.e., noninflammatory) conditions, which, however, was not significant. These findings are consistent with our previous reports where the in vitro differentiation potential of OB progenitors from *Ptx3^−/−^* mice was comparable to that of their WT counterparts [8]. However, in the same study, we reported that OB lineage cells had reduced activity in vivo. Arguably, one could speculate that microenvironmental or systemic cues are responsible for the defective osteogenesis observed in vivo but not in vitro in the absence of PTX3.

OB-derived PTX3 likely accumulates not only in the fluid phase (i.e., cell culture supernatant) but also in the extra- and pericellular matrix that is deposited by OBs upon osteogenic induction [39]. In this regard, it is known that PTX3 incorporates into HA-rich matrices through the action of the HA-binding protein TSG-6 and the serum proteoglycan IαI [4,6]. In the present work, we did not observe significant differences between *Ptx3^−/−^* and WT OBs in terms of expression of genes involved in HA synthesis. This observation somewhat conflicts with a report by Dong et al., where PTX3 was proposed to promote *Has2* and *Has3* transcription and HA deposition [18]. However, in that study, short/small hairpin RNA (shRNA) and lentiviral infections were used to manipulate the mRNA levels, which could arguably achieve only partial effects on the expression of the *Ptx3* gene. As opposed to this, in the present work, we exploited a clearcut strategy where complete abrogation of PTX3 expression was achieved through gene modification.

In our model, inflammation (i.e., TNF-α stimulation) impaired mineralization in both WT and *Ptx3^−/−^* OBs to a comparable extent; however, collagen production and *Has2* expression were reduced in *Ptx3^−/−^* (compared to WT) OBs, suggesting that in inflammatory conditions, PTX3 is required for effective synthesis and deposition of the organic components of the OB extracellular matrix that comprise HA in addition to collagen. In this regard, HA crosslinking by PTX3 was proposed as a protective mechanism that counteracts extracellular matrix alterations during inflammation [18,38], and Has2 is known to catalyze HA synthesis at a faster rate than other HA synthases and make high-molecular-weight hyaluronan chains [40]. These processes are likely impaired in *Ptx3^−/−^* OBs, and the increased expression of CD44 that we observed in these cells could be an attempt to overcome this defect. In fact, in the absence of PTX3, the extracellular network of HA loses stability and affinity/avidity for its major cellular receptor CD44, a process that could be counteracted, at least in part, by enhanced surface expression of CD44.

Importantly, we documented here that *Ptx3^−/−^* OBs had increased osteoclastogenic potential (i.e., RANKL/OPG ratio) compared to their WT counterparts. In line with this, *Ptx3^−/−^* OBs led to the formation of more TRAP^+^ cells (i.e., OCs) when cocultured with WT OC progenitors. These findings are consistent with our previous report showing that *Ptx3* gene inactivation in the mouse is associated with reduced trabecular bone volume during bone turnover and delayed callus mineralization in experimental fracture [8]. However, they conflict with previous observations by Lee et al. suggesting that PTX3 can stimulate osteoclastogenesis by increasing RANKL production in OBs [14]. This discrepancy might be attributed to the fact that Lee et al. used pre-OBs as a source of PTX3 (as opposed to mature OBs) and the recombinant protein to modulate PTX3 concentration in the model (in place of gene modification). Also, we did not see differences between *Ptx3^−/−^* and WT OBs in the RANKL/OPG ratio upon TNF-α stimulation during mineralization, perhaps suggesting that PTX3 is dispensable for TNF-α to exert its osteoclastogenic functions [41].

In the extracellular matrix, PTX3 interacts with several molecules; among them, TSG-6 has a role in extracellular matrix stability, and in bone, it inhibits osteoclastogenesis and OC activity by interacting with OPG [14]. In our experimental setting, *Tnfaip6* expression was comparable in *Ptx3^−/−^* and WT OBs in the absence of TNF-α with a trend of increased expression in *Ptx3^−/−^* OBs in the inflammatory setting. Moreover, we observed enhanced expression of CD44 on *Ptx3^−/−^* osteoblastic cells, which might amplify their adhesion to monocytic cells (through ICAM-1 and VCAM-1), whereby these cell-to-cell interactions were proposed to support osteoclastogenesis [42].

OBs are a source of cytokines and chemokines with essential roles in bone metabolism, including CCL2, CCL3, CCL5, CXCL9, IL-6, TNF-α, and TGF-β [20,43]. We investigated the effect of PTX3 genetic depletion on the expression of these genes and the synthesis of the corresponding proteins both in homeostatic and inflammatory conditions. Significant differences were observed between *Ptx3^−/−^* and WT OBs in terms of synthesis of the CCL2, CCL3, and IL-6 proteins, especially in the presence of TNF-α, despite the fact that the corresponding mRNA levels were not affected by the genetic ablation of PTX3. CCL2, CCL3, and IL-6 are key players in bone pathophysiology and potent modulators of the OB/OC balance [24]. It is thus likely that PTX3, mostly in inflammatory conditions, participates in bone metabolism through the control of synthesis and the release of these inflammatory mediators in addition to the modulation of RANKL and OPG production and the expression of extracellular matrix receptors and components. Also, CCL2 and CCL3 are powerful chemoattractants and modulators of leukocytes, mostly monocytes and neutrophils [44]. Based on our observations, it is therefore conceivable that PTX3 might be involved in the activation and recruitment of immune cells to the inflamed bone.

**Figure 7:**
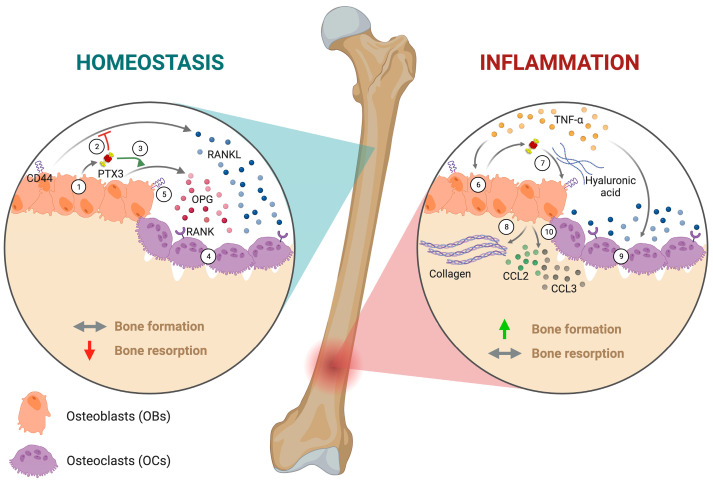
Schematic representation of the mechanism of Ptx3 regulation of OB and OC formation and function in homeostatic and inflammatory conditions. Left: In conditions of bone homeostasis, PTX3 is synthesized by pre- and mature OBs (1); the released protein inhibits RANKL production (2) and stimulates OPG release (3). This results in reduced RANKL/OPG ratio, which helps restraining osteoclastogenesis (4). A similar effect might be contributed to by the PTX3-dependent control of CD44 expression on OBs (5). Right: Inflammatory conditions (i.e., TNF-α stimulation) increase PTX3 expression in OBs (6). The newly synthesized protein, possibly via the HA network and the engagement of CD44 (7), stimulates collagen production and deposition by OBs (8). At the same time, TNF-α has direct osteoclastogenic effects on OC precursors (9). Also, PTX3 is required for OBs to make and release inflammatory chemokines like CCL2 and CCL3 (10). Created with BioRender.com.

Building upon our findings, we propose a role for PTX3 in the bone microenvironment where this pentraxin takes part in matrix organization and remodeling with diverse mechanisms in homeostatic and inflammatory settings (Figure 7). 

In conditions of bone homeostasis, PTX3 is synthesized by pre- and mature OBs (1), and the released protein, via unknown mechanisms, inhibits RANKL productions (2) while stimulating OPG release (3). This (i.e., reduced RANKL/OPG ratio) results in the inhibition of osteoclastogenesis (4). Such antiosteoclastogenic activity might also be contributed to by the PTX3-dependent control of CD44 expression on OBs (5). Inflammatory conditions (i.e., TNF-α stimulation) induce PTX3 overexpression in OBs (6); the newly synthesized protein, possibly via the HA network and the engagement of CD44 (7), stimulates collagen production and deposition by OBs (8), whereby TNF-α has direct osteoclastogenic effects on OC precursors (9). Also, in these conditions, PTX3-competent OBs synthesize and release inflammatory mediators that further contribute to the OB–OC crosstalk, including CCL2 and CCL3 (10). Based on this model, PTX3 exerts a net osteogenic effect on the OB/OC balance via the restraint of osteoclastogenesis in homeostatic conditions and the promotion of bone formation in inflammatory settings. 

In conclusion, our data point to PTX3 as an active player in the complex network of cellular and molecular processes that finely control bone metabolism in physiological and pathological conditions. Further investigations are needed to fully elucidate the translational relevance of these findings.

## 4. Materials and Methods

### 4.1. Generation of Primary Osteoblast Cultures

The upper part of the skull (denominated calvarium) was dissected from C57BL/6J wild-type (WT) and *Ptx3^−/−^* newborn mice (postnatal day 3–4), cleaned from adherent soft tissues, and then sequentially digested in 1× HBSS (EuroClone S.p.A, Pero, Italy.) supplemented with 1 mg/mL collagenase type IV (Sigma-Aldrich, St. Louis, MO, USA) and 0.025% trypsin (EuroClone S.p.A.) at 37 °C for 30 min in agitation. Digestion was repeated 4 times. The cells from digests 2–4 were pulled and plated in 6-well plates (6 × 10^5^ cells/well) in OB medium (α-MEM, 10% fetal bovine serum, FBS, and 1% penicillin/streptomycin, P/S).

The mice were maintained in the Specific-Pathogen-Free (SPF) Animal Facility of Humanitas Research Hospital (Rozzano, Milan, Italy). All the procedures were in accordance with the ethical rules of the Institutional Animal Care and Use Committee of Humanitas Research Hospital in compliance with national and international law and policies.

### 4.2. Osteogenic Differentiation

An amount of 1.5 × 10^5^ cells/well was plated in 24-well plates in OB medium and at 80% confluence cultured in osteogenic induction medium (oim) consisting in OB medium supplemented with 100 nM dexamethasone, 50 µM ascorbic acid, and 10 mM β-glycerophosphate (Sigma-Aldrich). In specific experiments, 10 ng/µL murine TNF-α was added to the medium to mimic inflammatory stimulation. The medium was changed twice a week.

### 4.3. Collagen Production

An amount of 1.7 × 10^5^ cells/well was plated in 12-well plates in OB medium and at 80% confluence treated with 50 µg/mL ascorbic acid for 8 h to stimulate collagen production. Afterwards, the cells were fixed with 4% PFA for 20 min at room temperature (RT), stained with Sirius red 0.1% in picric acid, and washed with phosphate-buffered saline, PBS. The stain was extracted with 300 µL of destain solution (0.2 M NaOH/MeOH 1:1), and the optical density was read at 540 nm on a Promega™ GloMax^®^ Plate Reader (Madison, WI, USA).

In selected experiments, OBs were treated with 10 ng/µL murine TNF-α for 24 h prior to stimulation with ascorbic acid.

### 4.4. Mineralization Capacity

After 7, 14, and 21 days of osteogenic induction, the culture medium was discarded and cells were fixed with 70% EtOH for 1 h at RT, then stained with Alizarin red (ARS) for 30 min at RT. The stain was extracted with acetic acid 10%, and absorbance was measured at 405 nm on a Promega™ GloMax^®^ Plate Reader.

### 4.5. Gene Expression Analysis

At 0, 7, 14, and 21 days of osteogenic induction, total RNA was extracted using Trizol (Qiagen, Hilden, Germany). Reverse transcription was carried out on 1.0 μg total RNA using a High-Capacity cDNA Reverse Transcription Kit (Applied Biosystems, Waltham, MA, USA) according to the manufacturer’s instructions. Quantitative PCR was performed on a ViiA7 Real-Time PCR Detection System (Applied Biosystems) using the SsoAdvanced™ SYBR^®^ Green Supermix (Bio-Rad, Waltham, MA, USA) following the manufacturer’s protocol. The *Gapdh* gene was used as a housekeeping gene. The sequence of the primers used are available upon request.

The relative gene expression analysis of target genes was conducted following the comparative 2^−ΔCt^ method, and the normalized gene expression was represented as arbitrary units (AU).

### 4.6. Osteoclastogenic Capacity

WT and *Ptx3^−/−^* OBs were cultivated in OB medium for 24 h. The day after, 1 × 10^6^ bone marrow cells from the femur of 2-month-old WT mice were plated on the OB monolayer. The coculture was maintained for 7 days in OB medium supplemented with 10 nM vitamin D and 1 µM prostaglandin E2 (PGE2)_._ Medium was changed twice a week. At the end, osteoclast formation in the cocultures was evaluated through TRAP staining following the manufacturer’s protocol (Sigma-Aldrich). TRAP^+^ multinucleated (nuclei number ≥ 3) OCs were counted by a blind operator and expressed as the number of mature OCs per well.

In parallel, RNA samples and conditioned medium were collected to perform qPCR and ELISA assays, respectively.

### 4.7. Enzyme-Linked Immunosorbent Assay (ELISA)

At different timepoints during osteogenic induction as well as in the coculture system, supernatants were collected to quantify PTX3, RANKL, and OPG using a dedicated commercial ELISA kit (R&D System, Minneapolis, MN, USA or ThermoFisher, Waltham, MA, USA). Briefly, collected supernatants were centrifuged for 5 min at RT and stored at −80 °C until use. ELISAs were performed following the manufacturer’s protocol of each kit, and the absorbance was read on a Promega™ GloMax^®^ Plate Reader.

### 4.8. Fluorescence-Activated Cell Sorting (FACS) Analysis

WT and *Ptx3^−/−^* OBs were cultured in OB medium until confluence, and the cells were detached by EDTA/trypsin and stained in PBS FACS (PBS with 5% FBS and 1 mM EDTA) with the following anti-mouse antibodies: CD44 BV650, Sca-1 BV711, CD51 biotinylated with APC-conjugated streptavidin, CD45 PerCP5.5, and Aqua Live/Dead (all BD). Samples were acquired on an LSR Fortessa flow cytometer (BD Biosciences; Franklin Lake, NJ, USA) and analyzed with FlowJo software, v. 10 (Tree Star, Inc; Ashland, OR, USA). At least 500,000 events were considered as a cutoff for FACS analysis.

### 4.9. Western Blotting

At 0, 7, and 14 days of osteogenic induction in the absence and presence of TNF-α, WT OBs were lysed in a commercial cell lysis buffer (Cell Signaling, Danvers, MA, USA) supplemented with proteases inhibitors (Sigma-Aldrich) and phosphatase inhibitors (ThermoFisher) according to the manufacturer’s instructions. The presence of PTX3 was assessed by Western blotting with chemiluminescence detection. Briefly, cell lysate aliquots containing 10 μg total proteins were denatured and reduced by heating at 70 °C for 10 min in sample loading buffer containing dithiothreitol (DTT, Life Technologie; Carlsbad, CA, USA). Proteins were separated by polyacrylamide-gel electrophoresis in the presence of sodium dodecyl sulfate (SDS-PAGE) on NuPAGE 4–12% Bis-Tris gels (Life Technologies) using MOPS SDS running buffer and transferred onto 0.45 μm polyvinylidene fluoride (PVDF) membranes. PTX3 was detected with an anti-murine PTX3 goat polyclonal antibody (R&D Systems; 25 ng/mL) followed by a secondary anti-goat IgG horseradish peroxidase (HRP) conjugate (1:5000 dilution; GE Healthcare, Chicago, IL, USA). Chemiluminescence was recorded on a Chemidoc system (Bio-Rad) following the addition of Immobilon Western Chemiluminescent HRP Substrate (Merck Millipore; Burlington, MA, USA).

### 4.10. Immunoassays

The concentrations of IL-6, TNF-α, CCL2, and CCL3 in the lysates of WT and *Ptx3^−/−^* murine OBs at 0, 7, and 14 days of osteogenic induction in the absence and presence of TNF-α were determined using the Ella™ Automated Immunoassay System (Bio-Techne; Minneapolis, MN, USA) following the manufacturer’s instructions.

### 4.11. Statistical Analysis

Statistical analysis and graphical representation of data were carried out using GraphPad Prism version 9.0 (GraphPad Software). Data were expressed as mean ± standard error of the mean (SEM). One- or two-way ANOVA with Tukey multiple comparison tests or Mann–Whitney tests for nonparametric data were used to calculate *p*-values. Significance is represented as follows: * *p* < 0.05, ** *p* < 0.01, *** *p* < 0.001.

## Data Availability

Data supporting the reported results can be found at https://zenodo.org/communities/humanitasirccs and will be made available upon request to the corresponding authors.

## Abbreviations

PTX3	pentraxin 3
OB	osteoblast
IL-1β	interleukin-1β
TNF-α	tumor necrosis factor-α
PRM	pattern recognition molecule
PAMPs	pathogen-associated molecular pattern
TSG-6	tumor necrosis factor-stimulated gene 6
IαI	inter-alpha-trypsin inhibitor
FGF2	fibroblast growth factor 2
Runx2	runt-related transcription factor 2
SP7	Sp7 transcription factor
Bglap	bone gamma-carboxyglutamate protein
FAK/Akt	focal adhesion kinase/serine/threonine kinase 1
BMSCs	bone marrow-derived stromal cells
oim	osteogenic induction medium
RANKL	Receptor Activator of Nuclear Factor Kappa B Ligand
OPG	osteoprotegerin
TRAP	tartrate-resistant acid phosphatase
Alp	alkaline phosphatase
Spp1	secreted phosphoprotein 1
Col1a1	collagen type I alpha 1 chain
OC	osteoclast
M-CSF	macrophage colony-stimulating factor
RANK	receptor activator of nuclear factor κ B
VCAM1	vascular cell adhesion molecule 1
HAS	hyaluronan synthase
ICAM1	intercellular adhesion molecule 1
Ccl3	C-C motif chemokine ligand 3
Ccl5	C-C motif chemokine ligand 5
Cxcl9	C-X-C motif chemokine ligand 9
TGF-β	transforming growth factor-beta
IL-6	interleukin 6
CCL2	C-C motif chemokine ligand 2
ARS	Alizarin red staining
